# Supplementary material for: Apolipoprotein L9 interacts with LC3/GABARAP and is a microtubule-associated protein with a widespread subcellular distribution
Source: Biol Open. 2019 Sep 12;8(9):bio045930. doi: 10.1242/bio.045930 (PMC6777357; doi:10.1242/bio.045930)
Supplement: Supplementary information [file biolopen-8-045930-s1.pdf]

Figure S1

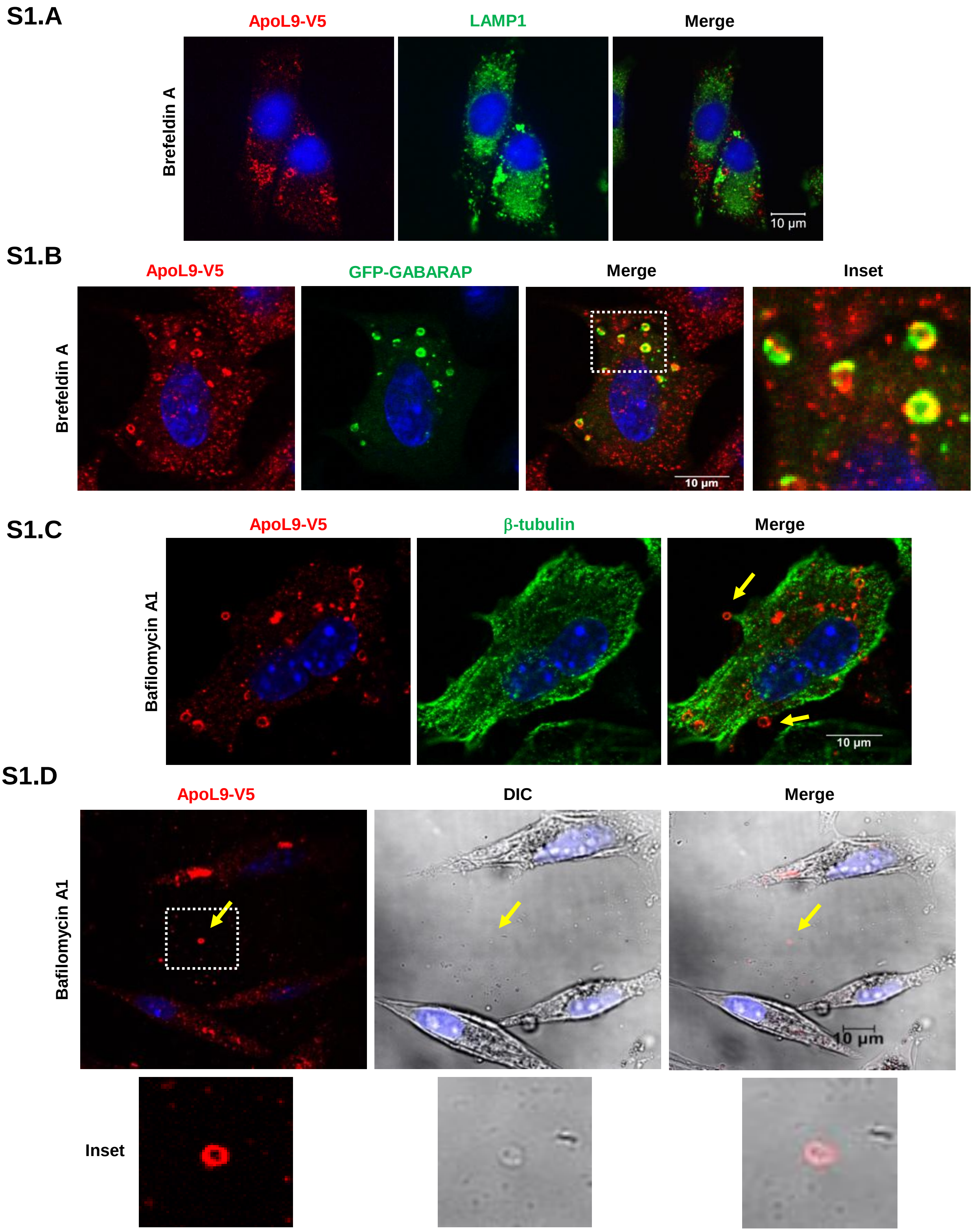

Biology Open • Supplementary information

Fig.S1:

A. Indirect immunofluorescence for ApoL9-V5 and LAMP1 in B16F10^{L9} cells treated with 10 mg mL^{-1} brefeldin A for 16 h. Note the lack of co-localization of ApoL9 with LAMP1. **B.** Indirect immunofluorescence for ApoL9-V5 and GFP-GABARAP in B16F10^{L9} cells treated with 10 mg mL^{-1} brefeldin A for 16 h. Note the presence of GABARAP on/near all structures (mitochondria) positive for ApoL9. Boxed inset is magnified for better viewing. **C.** Ring-like structures positive for ApoL9-V5 exiting a cell treated with bafilomycin A1 for 16 h (yellow arrows). Staining for tubulin (green) helps visualize the cell boundary. **D.** Released ring-like structure in the extracellular region (yellow arrow) and magnified boxed inset. Note the vesicle-like appearance of the same in the DIC image.

Biology Open • Supplementary information

Figure S2

S2.A

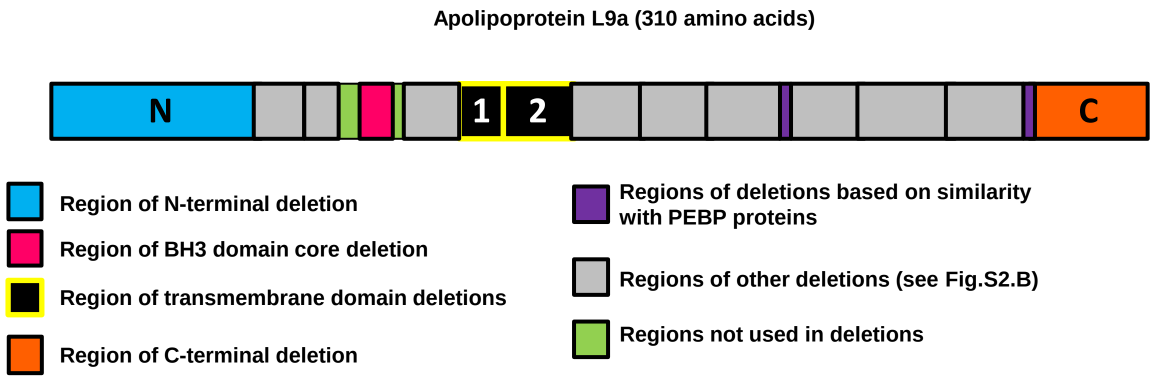

S2.B

Deletion	Residues	Reasoning behind deletion	Deletion	Residues	Reasoning behind deletion
ΔN	1-54	N-terminal helices	Δ166-186	166-186	Random deletion
Δ51-63	51-63	Random deletion	Δ187-206	187-206	Random deletion
Δ64-74	64-74	Random deletion	Δ207-209	207-209	Conserved identity with human PEBP4
ΔBH3	81-89	Core BH3 consensus	Δ210-230	210-230	Random deletion
Δ92-110	92-110	Random deletion	Δ231-254	231-254	Random deletion
Δ111-123 (TM1)	111-123	Part of predicted TM domain	Δ255-276	255-276	Random deletion
Δ124-144 (TM2)	124-144	Part of predicted TM domain	Δ277-279	277-279	Conserved identity with mouse PEBP1
Δ145-165	145-165	Random deletion	ΔC	281-310	Predicted coiled coil domain

S2.C

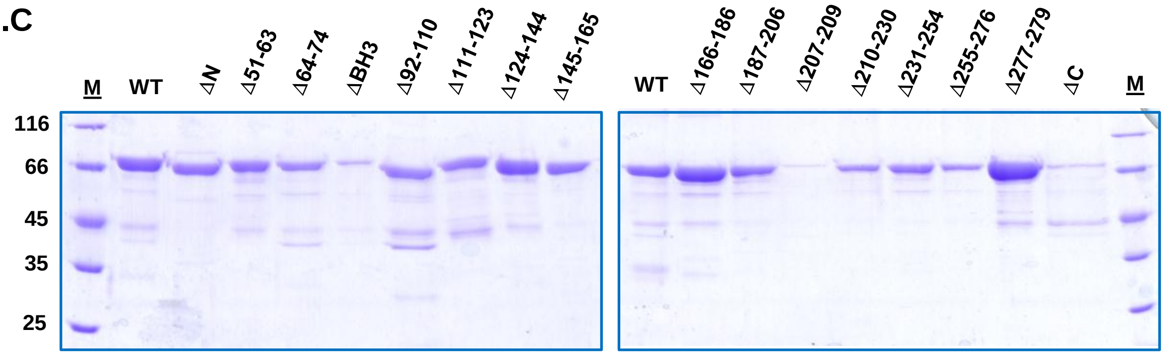

Fig.S2:

A. Schematic of deletions constructed in the ApoL9 coding sequence to screen for PE-binding. **B.** Table detailing the deletions made in the ApoL9 coding sequence and the reasoning behind the various deletions. **C.** MBP-ApoL9 deletion mutant proteins resolved by SDS-PAGE and stained with Coomassie Brilliant Blue.

Biology Open • Supplementary information

Figure S3

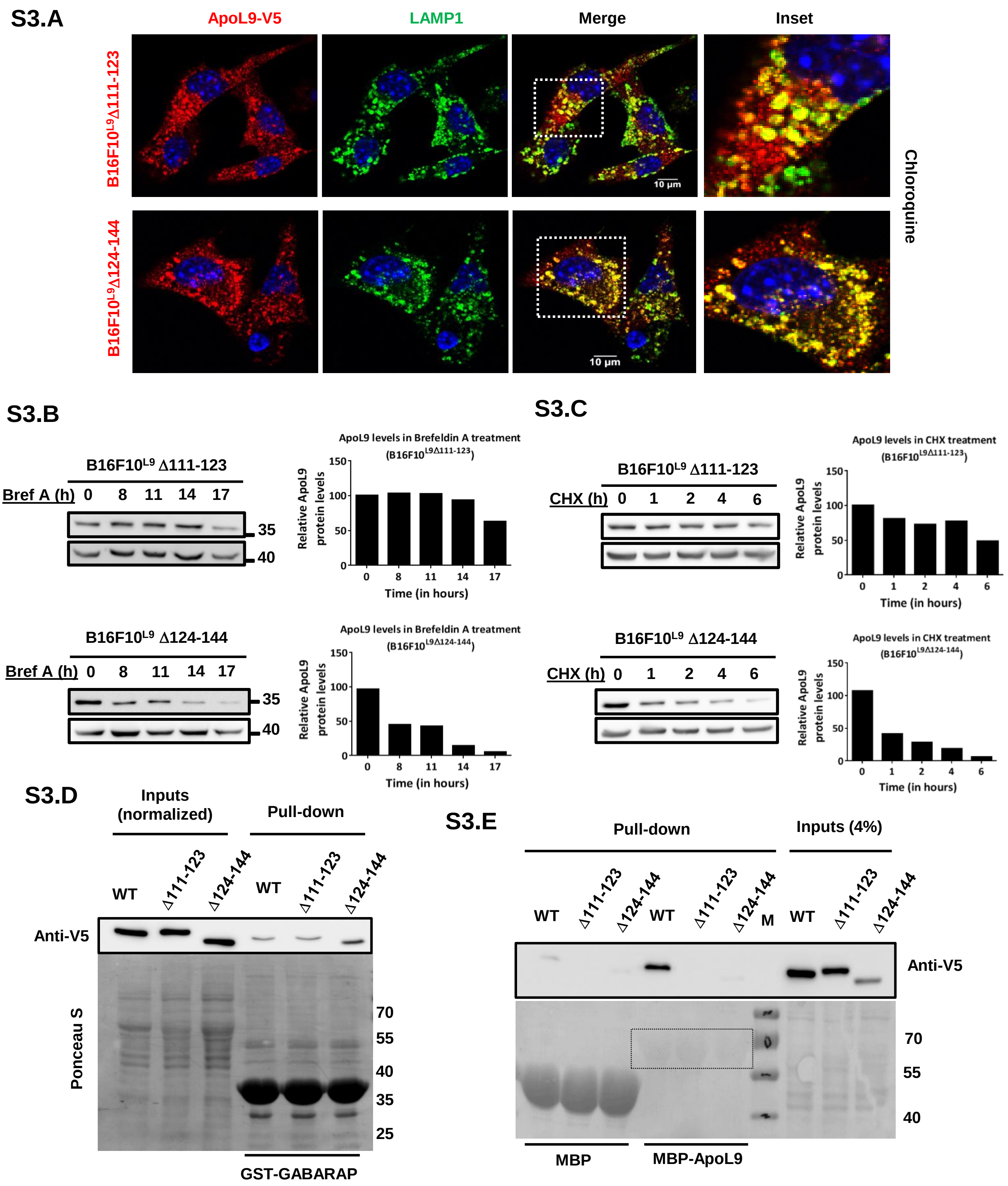
S3.A
ApoL9-V5
LAMP1
Merge
Inset
B16F10L9Δ111-123
B16F10L9Δ124-144
Chloroquine
10 µm
S3.B
B16F10L9 Δ111-123
Bref A (h) 0 8 11 14 17
35
40
ApoL9 levels in Brefeldin A treatment (B16F10L9Δ111-123)
Relative ApoL9 protein levels
Time (in hours)
B16F10L9 Δ124-144
ApoL9 levels in Brefeldin A treatment (B16F10L9Δ124-144)
S3.C
B16F10L9 Δ111-123
CHX (h) 0 1 2 4 6
ApoL9 levels in CHX treatment (B16F10L9Δ111-123)
B16F10L9 Δ124-144
ApoL9 levels in CHX treatment (B16F10L9Δ124-144)
S3.D
Inputs (normalized)
Pull-down
WT
Δ111-123
Δ124-144
Anti-V5
Ponceau S
70
55
40
35
25
GST-GABARAP
S3.E
Pull-down
Inputs (4%)
M
MBP
MBP-ApoL9

Fig.S3:

A. Indirect immunofluorescence for ApoL9-V5 and LAMP1 in B16F10$^{L9\Delta111\text{-}123}$ and B16F10$^{L9\Delta124\text{-}144}$ cells treated with chloroquine for 12 hours. Boxed insets are magnified and shown. Anti-V5 and anti-LAMP1 antibodies were used. **B.** ApoL9-V5 levels in B16F10$^{L9\Delta111\text{-}123}$ and B16F10$^{L9\Delta124\text{-}144}$ cells treated with brefeldin A for the indicated time periods and (right) quantification of the same. Note the distinct decrease in ApoL9 levels only in B16F10$^{L9\Delta124\text{-}144}$. **C.** ApoL9-V5 levels in B16F10$^{L9\Delta111\text{-}123}$ and B16F10$^{L9\Delta124\text{-}144}$ cells treated with 50 mg mL^{-1} cycloheximide for the indicated time periods and (right) quantification of the same. Anti-V5 antibody was used for immunoblotting. **D.** Pull-down of ApoL9$^{\Delta111\text{-}123}$-V5 and ApoL9$^{\Delta124\text{-}144}$-V5 (from HEK293T lysates) by recombinant GST-GABARAP, similar to Fig. 1A. WT ApoL9-V5 binding to GST-GABARAP serves as a positive control. Levels of input were normalized such that equal quantities of mutant proteins were used in the assay. Recombinant GST-GABARAP is stained with Ponceau S. **E.** Pull-down of ApoL9$^{\Delta111\text{-}123}$-V5 and ApoL9$^{\Delta124\text{-}144}$-V5 (from HEK293T lysates) by recombinant MBP and MBP-ApoL9 bound to amylose resin. WT ApoL9-V5 binding to MBP-ApoL9 serves as a positive control. MBP and MBP-ApoL9 are shown stained by Ponceau S. Loading of MBP-ApoL9 is lesser than that of MBP (though in excess), hence it is shown boxed.

Biology Open • Supplementary information

Figure S4

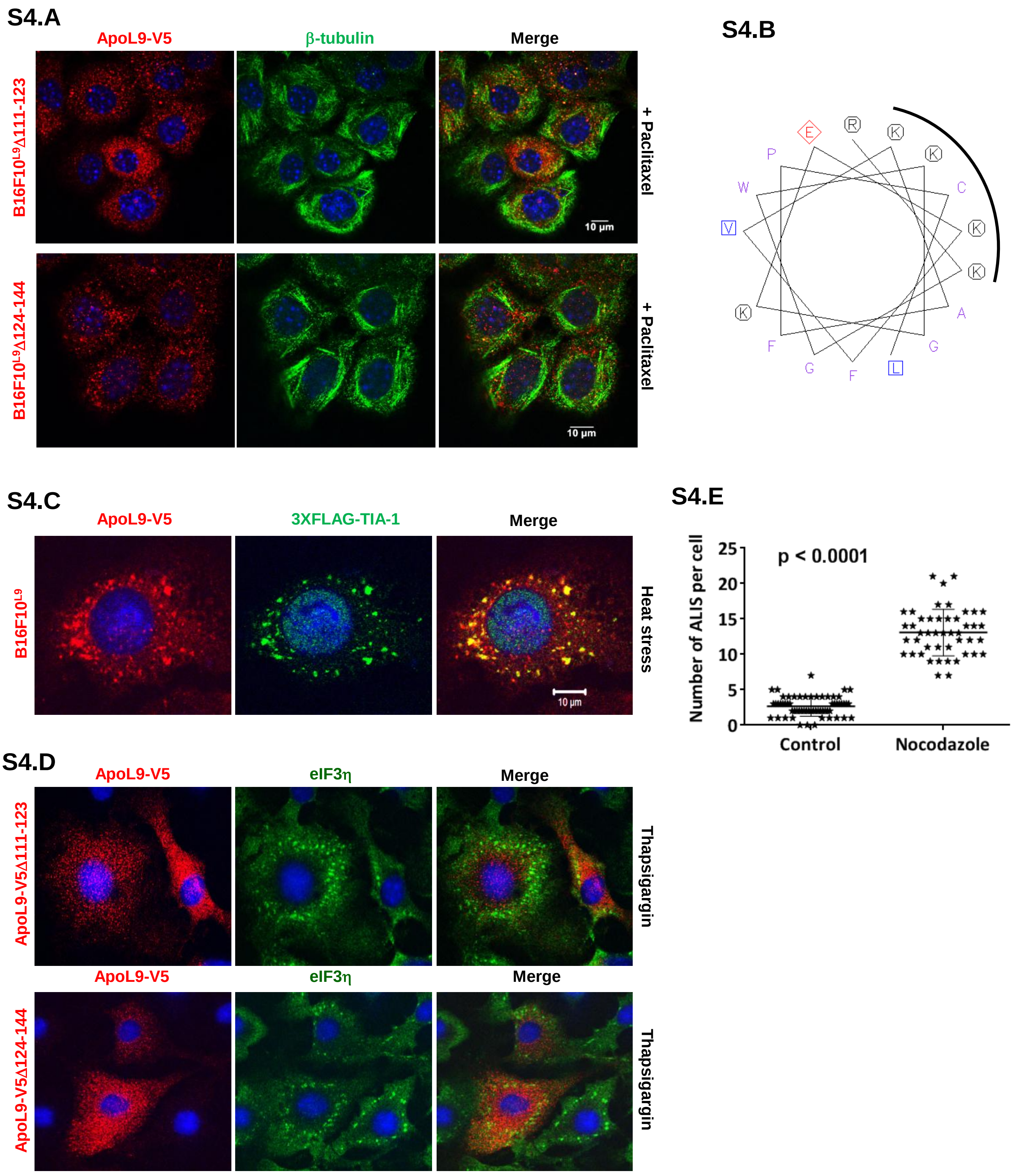

Biology Open • Supplementary information

S4.F

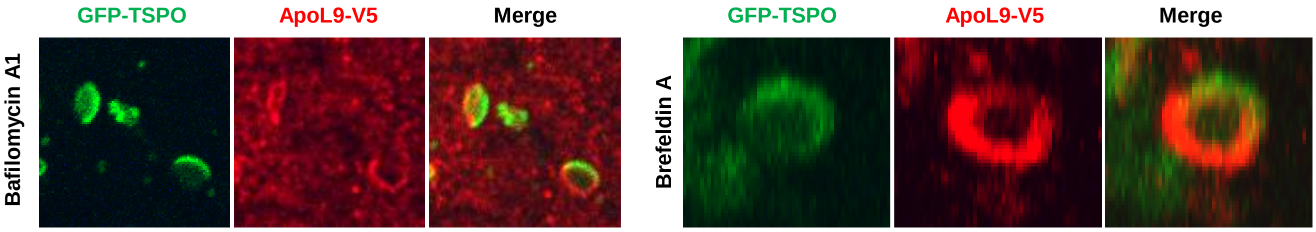

S4.G

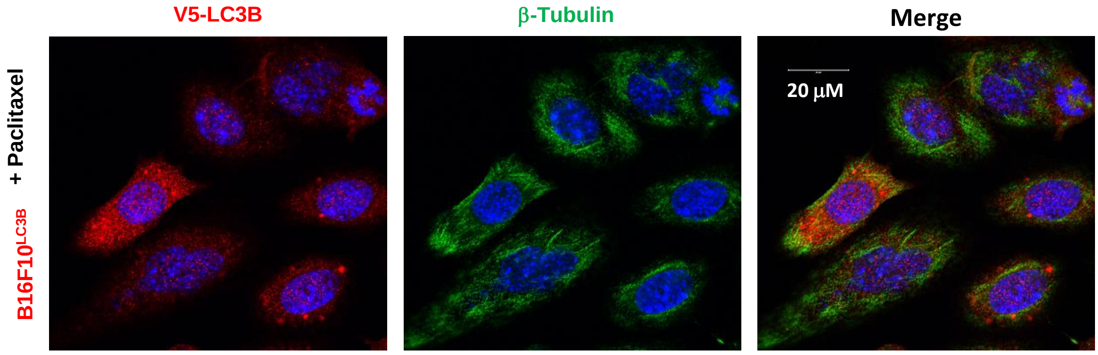

Fig.S4:

A. Indirect immunofluorescence for ApoL9-V5 and β-tubulin in B16F10$^{L9\Delta111\text{-}123}$ and B16F10$^{L9\Delta124\text{-}144}$ cells treated with paclitaxel for 10 h. Note the lack of association of ApoL9 with microtubules. **B**. The region of the helix comprising amino acids 54-70 of rat ApoL9a, viewed as a helical wheel at http://www.bioinformatics.nl/cgi-bin/emboss/pepwheel . Note the conserved lysines by comparing with mouse ApoL9a in Fig. 7H. **C.** Indirect immunofluorescence for ApoL9-V5 and stress granule marker 3XFLAG-TIA-1 in B16F10^{L9} cells treated at 43˚C for 30 min. The *3XFLAG-TIA-1* construct was electroporated into B16F10^{L9} cells. Anti-V5 and anti-FLAG antibodies were used. **D.** Indirect immunofluorescence for ApoL9$^{\Delta111\text{-}123}$-V5 and ApoL9$^{\Delta124\text{-}144}$-V5 and eIF3h in B16F10 cells electroporated with the respective ApoL9 deletion mutants and treated with 500 nM thapsigargin for 1 h. Neither of the mutants is present in stress granules. **E.** Quantification of ALIS in B16F10^{L9} cells with and without treatment with nocodazole (n > 40 cells). p-value determined by unpaired t-test. **F.** Magnified images of selected ApoL9-positive mitochondria in B16F10^{L9} cells treated with bafilomycin A1/brefeldin A. Note how parts of the mitochondria densely stained for TSPO-GFP are weakly stained for ApoL9-V5, and vice versa. **G.** Indirect immunofluorescence for V5-LC3B and β-tubulin in B16F10^{LC3B} cells treated with paclitaxel for 10 h. Note that LC3B is not evidently visible on microtubules (compare with Fig. 7E).
